# Risk factors and outcomes associated with type of uterine rupture

**DOI:** 10.1007/s00404-022-06452-0

**Published:** 2022-03-14

**Authors:** D. Dimitrova, AL. Kästner, AN. Kästner, A. Paping, W. Henrich, T. Braun

**Affiliations:** 1 Department of Obstetrics and Department of Gynecology With Center for Oncological Surgery, Charité–Universitätsmedizin Berlin, Corporate member of Freie Universität Berlin and Humboldt-Universität Zu Berlin, Augustenburger Platz 1, 13353 Berlin, Germany; 2 Department of Obstetrics, Charité–Universitätsmedizin Berlin, Corporate member of Freie Universität Berlin and Humboldt-Universität Zu Berlin, Augustenburger Platz 1, 13353 Berlin, Germany; 3 Department of Surgery, Charité–Universitätsmedizin Berlin, Corporate member of Freie Universität Berlin and Humboldt-Universität Zu Berlin, Augustenburger Platz 1, 13353 Berlin, Germany; 4 Department of Experimental Obstetrics, Charité–Universitätsmedizin Berlin, Corporate member of Freie Universität Berlin and Humboldt-Universität Zu Berlin, Augustenburger Platz 1, 13353 Berlin, Germany

**Keywords:** Complete uterine rupture, Cesarean delivery, Trial of labor after cesarean, Vaginal birth after cesarean, Elective repeat cesarean delivery, Feto-maternal outcome

## Abstract

**Purpose:**

To identify risk factors associated with the occurrence of complete uterine rupture (CUR) in comparison to partial uterine rupture (PUR) to further investigate to what extent a standardized definition is needed and what clinical implications can be drawn.

**Methods:**

Between 2005 and 2017 cases with CUR and PUR at Charité University Berlin, Germany were retrospectively identified. Demographic, obstetric and outcome variables were analyzed regarding the type of rupture. Binary multivariate regression analysis was conducted to identify risk factors associated with CUR. In addition, the intended route of delivery (trial of labor after cesarean delivery (TOLAC) and elective repeat cesarean delivery (ERCD)), divided according to the type of rupture, was compared.

**Results:**

92 cases with uterine rupture were identified out of a total of 64.063 births (0.14%). Puerperal complications were more frequent in CUR (67.9 versus 41.1%, *p* = 0.021). Multiparity ≥ 3 was more frequent in CUR (31 versus 10.7%, *p* = 0.020). Factors increasing the risk for CUR were parity ≥ 3 (OR = 3.8, *p* = 0.025), previous vaginal birth (OR = 4.4, *p* = 0.011), TOLAC (OR = 6.5, *p* < 0.001) and the use of oxytocin (OR = 2.9, *p* = 0.036). After multivariate analysis, the only independent risk factor associated with CUR was TOLAC (OR = 7.4, *p* = 0.017).

**Conclusion:**

TOLAC is the only independent risk factor for CUR. After optimized antenatal counselling TOLAC and ERCD had comparable short-term maternal and fetal outcomes in a high resource setting. A high number of previous vaginal births does not eliminate the risk of uterine rupture. A clear distinction between CUR and PUR is essential to ensure comparability among studies.

**Supplementary Information:**

The online version contains supplementary material available at 10.1007/s00404-022-06452-0.

## Introduction

Peripartum uterine rupture is a rare obstetric complication associated with a previous cesarean delivery often resulting in adverse maternal and neonatal outcomes. During the past decades an increase in uterine rupture rates was noted [1]. According to the German Obstetric Surveillance System (GerOSS), an organisation conducting prospective population-based studies of serious and rare disorders in pregnancy and childbirth for Lower Saxony only, the prevalence for uterine rupture is estimated at 3.2 per 10,000 deliveries in total, with previous cesarean delivery at 20.0, with previous cesarean delivery and labor at 27.0 and 0.4 per 10,000 deliveries without previous cesarean section [2].

The strongest risk factor for a uterine rupture is trial of labor after cesarean (TOLAC)-irrespective of the final birth mode, but also influenced by other factors, such as interdelivery interval shorter than 16 months, induction of labor with prostaglandins and oxytocin, augmentation of labor with oxytocin, birthweight, gestational age and some maternal characteristics, such as age ≥ 35 years, height ≤ 164 cm and parity ≥ 3 [3–5]. Landon et al. also stated in 2004 that TOLAC is associated with a greater perinatal risk, such as the risk of stillbirth, neonatal death, or hypoxic–ischemic encephalopathy compared to an elective repeated cesarean section (ERCD) [6]. This could lead to the assumption to primarily recommend all women with a previous caesarean section in the proceeding pregnancy an ERCD. On the other hand, ERCDs are associated with increased risks for perioperative complications such as severe postpartum haemorrhage and long-term complications such as abnormal invasive placentation or uterine diverticulum niche with reduced fertility [7–12].

Current TOLAC practice guidelines from the American College of Obstetricians and Gynecologists (ACOG) recommend offering TOLAC to women with one previous cesarean delivery and a low-transverse incision [13]. The chance for a successful TOLAC is higher for women who have had previous vaginal deliveries including previous vaginal births after cesarean (VBAC) (OR 3.9; 95% CI 3.6–4.3). It is lowered by labor induction and maternal obesity [14]. However, Wingert et al. stated recently that there is insufficient high-quality evidence for optimal pharmacologic and non-pharmacologic intervention for labor induction among women attempting a trial of labor after prior cesarean delivery [15].

The selection of low-risk candidates for uterine rupture during TOLAC remains crucial. There are several critical points, which have not been addressed so far. No standardized definition of uterine rupture has been established. Previous studies have mainly examined risk factors for complete uterine rupture or made no specific distinction between the type of uterine rupture, resulting in several problems: e.g., the incidence of uterine ruptures may be underestimated and identified risk factors for uterine ruptures might be rather applicable to women with CUR rather than PUR. Therefore, it is not yet clear, which factors lead a woman to develop a CUR more likely than a PUR.

Thus, the primary aim of this study was to compare the outcome between CUR and PUR, to identify risk factors associated with CUR and to further investigate to what extent a standardized definition is needed to provide a better risk estimation and saver birth planning for woman in the following pregnancies after previous caesarean delivery. Second, to compare risk factors and outcomes regarding the intended route of delivery (TOLAC with ERCD) in women with uterine rupture.

## Methods

### Study population

Medical records of cases with CUR or PUR between January 2005 and December 2017 at the Department of Obstetrics, Charité University Berlin, Germany were retrospectively identified. Approval for this study was obtained from the Ethics Commission of Charité–Universitätsmedizin Berlin (EA2/013/18).

### Definition of uterine rupture

A CUR was defined as the complete disruption of all uterine wall layers, including uterine serosa with free connection to the peritoneal cavity during pregnancy or delivery, irrespective of symptoms [16, 17]. PUR was defined as a wall dehiscence of the uterus, whereof the serosa is unaffected [16, 17].

Cases of CUR and PUR were identified and classified according to the information available from the surgical reports of the cesarean delivery.

### Data collection

All data were collected from our data base at the Charité–Universitätsmedizin Berlin. *Demographic variables* included maternal age in years at time of delivery (< 35 years versus ≥ 35 years [5]), height in cm (≤ 160 cm versus > 160 cm [18]), weight in kg at time of delivery and body mass index (BMI in kg/m^2^, grouped as ≤ 30 versus > 30 kg/m^2^ [18]). *Obstetric variables* included gravidity and parity (only considering children weighing > 500 g, grouped as < 3 versus ≥ 3 [5]), previous vaginal births, miscarriages, terminations of pregnancy, dilatation and curettages, ectopic pregnancies, cesarean sections and myoma operation. Furthermore, the diagnosis of gestational diabetes mellitus and hypertensive disorders during pregnancy was documented*. *Labor characteristics and outcomes included gestational age in weeks, medical labor induction and augmentation, the use of prostaglandins or oxytocin, the use of regional anaesthesia, symptoms of uterine rupture, such as severe pain at the LUS, hemodynamic problems or a pathological CTG as well as the planned and final delivery route.

Short-term maternal outcomes were peripartum hysterectomy, the need of blood transfusion, puerperal complications and maternal mortality. Documented neonatal outcomes were gender (male versus female), birth weight (< 3500 versus ≥ 3500 g), neonatal acidosis (cord blood pH < 7.2), severe neonatal acidosis (cord blood pH < 7.0), 5-min APGAR-Score (< 7 versus ≥ 7), the occurrence of hypoxic–ischemic encephalopathy and perinatal mortality. Intrauterine death after 24 completed weeks of gestation was defined as stillbirth, whereas perinatal mortality was defined as stillbirths and early neonatal deaths (up to 7 days of life). Multiple pregnancies were not included in the neonatal outcome analysis.

### Statistical methods

**Fig. 1:**
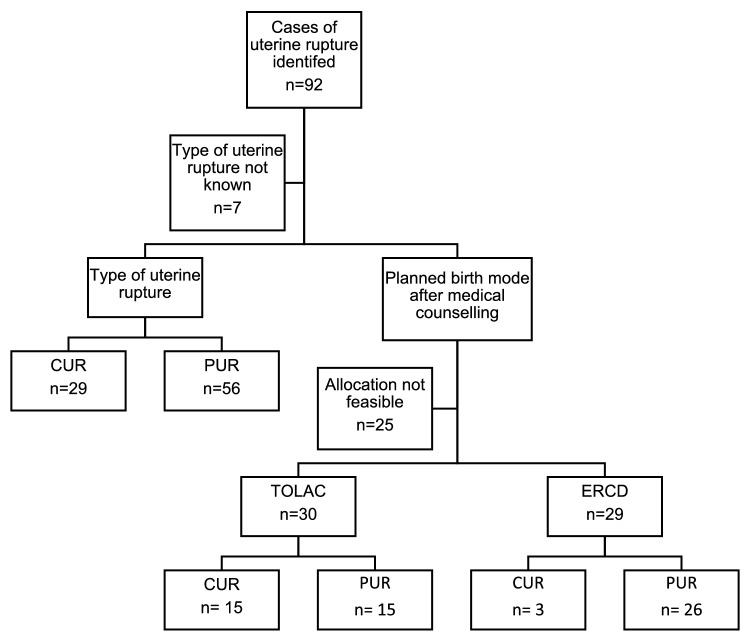
Analysis of uterine rupture cases. *CUR*, complete uterine rupture, *PUR*, partial uterine rupture, *TOLAC*, Trial of labor after cesarean, *ERCD*, elective repeat cesarean delivery

Groups were compared between type of rupture (CUR and PUR) and the intended route of delivery (TOLAC and ERCD), subdivided regarding type of uterine rupture (Fig. 1). For univariate analysis categorial variables were expressed as number or frequency (%) and analysed using Pearsons’s Chi-square test or Fisher’s exact test, as indicated. Continuous variables were tested for normal distribution using the Shapiro–Wilk test and were displayed as median with minimum and maximum or as mean with standard deviation. When normal distribution was ensured the *t* test was used, otherwise the Mann–Whitney *U* test was used to explore group differences. When no clear distinction between CUR and PUR was made, cases were not analysed.

Furthermore, a binary regression analysis (CUR versus PUR) was conducted to identify potential risk factors for a CUR. After univariate analysis, variables were only taken into consideration for multivariate analysis when statistical significance was given (*p* < 0.05). Because of missing data, multiple imputation with *n* = 5 was conducted before the multivariate analysis. Odds Ratios (OR) were presented with 95% confidence interval (95% CI). Statistical analyses were performed using statistical software package IBM SPSS Statistics, Version 23. *p* values < 0.05 were considered statistically significant.

## Results

During the study period of 12 years, 92 uterine ruptures have been identified, whereof 29 (31.5%) were CURs and 56 (60.9%) PURs (Fig. 1). In 7 cases (7.6%) no distinction between CUR and PUR could be made from the records and were, therefore, excluded from further analyses. There was a total of 64.063 births during the study period. The incidence of uterine ruptures, therefore, was 0.14% in total, 0.05% in cases of CUR and 0.09% in cases of PUR.

Patient’s characteristics are shown in Table 1. Multiparity ≥ 3 was seen in 31% (*n* = 9) in cases with CUR compared to 10.7% (*n* = 6) in cases with PUR (*p* = 0.020).

**Table 1:** Study cohort

	CUR (*n* = 29)	PUR (*n* = 56)	*p *value
Maternal age ≥ 35 years, *n* (%)	10 (34.5)	17 (30.4)	0.699^1^
Height ≤ 160 cm, *n* (%)	8 (29.6)	14 (25.5)	0.688^1^
BMI > 30 kg/m^2^, *n* (%)	11 (47.8)	15 (27.8)	0.089^1^
Parity ≥ 3, *n* (%)	9 (31.0)	6 (10.7)	**0.020**^1^
Previous vaginal birth, *n* (%)	10 (34.5)	6 (10.7)	**0.008**^1^
Previous VBAC, *n* (%)	4 (17.4)	2 (3.6)	0.059^2^
Previous cesarean delivery, *n* (%)	23 (79.3)	55 (98.2)	**0.006**^2^
Previous abortions/terminations of pregnancy ≥ 2, *n* (%)	3 (10.3)	11 (19.6)	0.363^2^
Previous myoma enucleation, *n* (%)*	3 (10.3)	4 (7.1)	0.686^2^
Unscarred uterus/spontaneous uterine rupture, *n* (%)	4 (13.8)	1 (1.8)	**0.044**^2^
Scarred uterus (previous cesarean delivery or myoma enucleation), *n* (%)	25 (86.2)	55 (98.2)	**0.044**^2^
Gestational diabetes mellitus, *n* (%)	1 (4.2)	9 (16.4)	0.268^2^
Pregnancy induced hypertension/Preeclampsia/Eclampsia/HELLP, *n* (%)	2 (6.9)	1 (1.8)	0.267^2^
Multifetal pregnancy, *n* (%)	2 (6.9)	1 (1.8)	0.267^2^
Placenta accreta spectrum, *n* (%)	4 (13.8)	1 (1.8)	**0.044**^2^

Significant results with *p*-value < 0.05

^1^Chi-square test

^2^Fisher’s exact test

^*^Four cases with previous myoma enucleation and PUR had also previous cesarean deliveries. From the 3 cases with CUR after myoma enucleation one had also previous cesarean delivery and 2 cases only myoma enucleations

Labor characteristics and delivery outcomes are reported in Table 2. In cases with CUR, TOLAC rates were significantly higher (*p* = 0.001) and elective cesarean delivery rates were significantly lower (*p* = 0.002) compared to cases with PUR.

**Table 2:** Labor characteristics compared between cases with CUR and PUR

	CUR (*n* = 29)	PUR (*n* = 56)	*p *value
Gestational age in weeks, *median (range)*	40 (23–42)	39 (34–44)	0.193^3^
Induction of labor, *n* (%)*	4 (17.4)	2 (6.9)	0.387^2^
* Prostaglandin, n (%)*	*1 (25)*	*1 (50)*	
* Oxytocin, n (%)*	*3 (75)*	*1 (50)*	
Augmentation with Oxytocin, *n* (%)*	9 (39.1)	10 (34.5)	0.730^1^
Regional anesthesia, *n* (%)	19 (65.5)	49 (89.1)	**0.009**^**1**^
Pathological CTG, *n* (%)	16 (55.2)	16 (29.1)	**0.019**^**1**^
TOLAC, *n* (%)	16 (66.7)	15 (26.8)	**0.001**^**1**^
*VBAC*, *n* *(%)*	*1 (6.3)*	*0 (0)*	
Cesarean delivery, *n* (%)	27 (93.1)	56 (100)	0.114^2^
Elective cesarean delivery, *n* (%)	*4 (14.8)*	*28 (50)*	**0.002**^**1**^
Vaginal birth, *n* (%)	2 (6.9)	0 (0)	0.114^2^
Symptomatic uterine rupture, *n* (%)	**26 (89.7)**	**26 (46.4)**	**< 0.001**^**1**^
*Pain at the LUS*, *n (%)*	*8 (30.8)*	*10 (38.5)*	
*Pathological CTG*, *n (%)*	*16 (61.5)*	*16 (61.5)*	
*Maternal hemodynamic problems*, *n (%)*	*2 (7.7)*	*0 (0)*	
Atony, *n* (%)	**10 (35.7)**	**3 (6.1)**	**0.001**^**2**^
*Atony I° with 500–1000 ml blood loss*, *n (%)*	*0 (0)*	*1 (33.3)*	
*Atony II° with 1001–1500 ml blood loss*, *n (%)*	*4 (40)*	*2 (66.7)*	
*Atony III° with > 1500 ml blood loss*, *n (%)*	*6 (60)*	*0 (0)*	

Significant results with *p*-value < 0.05

^1^Chi square test

^2^Fisher’s exact test

^3^Mann–Whitney *U* test

^*^Data related to all women without elective cesarean (CUR: *n* = 23, PUR: *n* = 29)

As expected, symptomatic uterine rupture occurred with 89.7% (*n* = 26) significantly more often in cases with CUR, manifesting as pain at the lower uterine segment (LUS), pathological CTG or maternal hemodynamic problems compared to cases with PUR in 46.4% (*n* = 26; *p* < 0.001). In cases with PUR, pathological CTG was seen in 29.1% (*n* = 16). Spontaneous ruptures without any previous uterine surgery occurred in 13.8% (*n* = 4) in the CUR-group compared to 1.8% (*n* = 1) in the PUR-group (supplemental Table 1).

The total postpartum hysterectomy rate was 4.7% (*n* = 4) in this study and no differences between the groups were observed (Table 3). In cases with CUR the need of maternal blood transfusions was significantly higher (CUR, *n* = 10, 34.5%; PUR, *n* = 2, 3.6%, *p* < 0.001) and puerperal complications were more frequent (CUR, *n* = 19, 67.9%; PUR, *n* = 23, 41.1%, *p* = 0.021) compared to cases with PUR. No maternal death occurred.

**Table 3:** Neonatal and maternal outcomes compared between cases with CUR and PUR

	CUR (*n* = 29)	PUR (*n* = 56)	*p *value
Maternal outcomes			
Postpartum hysterectomy, *n* (%)	3 (10.3)	1 (1.8)	0.113^2^
Maternal blood transfusion, *n* (%)	10 (34.5)	2 (3.6)	**< 0.001**^**2**^
Puerperal complications, *n* (%)	19 (67.9)	23 (41.1)	**0.021**^**1**^
*Fever* > *2d* > *38 °C postpartum, n (%)*	*4 (14.3)*	*2 (3.6)*	***0.092***^***2***^
*Impaired wound healing, n (%)*	*3 (10.7)*	*2 (3.6)*	*0.327*^*2*^
*Hypertension, n (%)*	*2 (7.1)*	*0 (0)*	*0.108*^*2*^
*Anemia (Hb* < *10 g/dl), n (%)*	*17 (60.7)*	*20 (35.7)*	***0.030***^***1***^
Maternal death, *n* (%)	0 (0)	0 (0)	1.0^1^
Neonatal outcomes^a^			
Male, *n* (%)	14 (48.4)	33 (60.0)	0.949^1^
Birth weight ≥ 3500 g, *n* (%)	11 (40.7)	19 (34.5)	0.584^1^
Cord blood pH < 7.2, *n* (%)	13 (52.0)	3 (5.6)	**< 0.001**^**1**^
Cord blood pH < 7.0, *n* (%)	7 (28.0)	0 (0)	**< 0.001**^**2**^
5-min Apgar score < 7, *n* (%)	7 (28.0)	1 (1.8)	**0.001**^**2**^
Need of intensive care, *n* (%)	11 (44.0)	5 (9.1)	**< 0.001**^**1**^
Hypoxic–ischemic encephalopathy, *n (%)*	2 (8.0)	0 (0)	0.116^2^
Perinatal mortality, *n* (%)	2 (8.0)	0 (0)	0.095^2^
* Mortality before hospital admission*, *n (%)*	*1 (50.0)*	*0 (0)*	

Significant results with *p*-value < 0.05

^1^Chi square test

^2^Fisher’s exact test

^a^After PUR: *n* = 55 (one case of twins excluded) and after CUR: *n* = 27 (one case of twins and one case of triplets excluded)

After CUR severe neonatal acidosis was seen in 28.0% (*n* = 7) compared to no cases after PUR (*p* < 0.001). A 5-min Apgar score < 7 was seen in 28.0% (*n* = 7) of the infants after CUR compared to 1.8% (*n* = 1) after PUR. The perinatal mortality was 7.4% (*n* = 2) in cases with CUR, whereof 50.0% died before hospital admission compared to nil cases with PUR (*p* = 0.106).

Factors increasing the risk for a CUR were parity ≥ 3 (OR = 3.8, *p* = 0.025), previous vaginal birth (OR = 4.4, *p* = 0.011), TOLAC (OR = 6.5, *p* < 0.001) and the use of oxytocin (OR = 2.9, *p* = 0.036; Table 7). At multivariate analysis, the only independent risk factor remaining associated with CUR was TOLAC (OR = 7.4, *p* = 0.017; Table 4).

**Table 4:** Univariate and multivariate binary logistic regression model for CUR

	Univariate		Multivariate	
	Crude OR (95% KI)	*p* value	Adjusted OR (95% KI)	*p* value
Parity > 3	3.750 (1.180–11.913)	**0.025**	4.568 (0.873–23.902)	0.072
Previous vaginal birth (Y/N)	4.386 (1.400–13.737)	**0.011**	0.882 (0.173–4.508)	0.880
Number of previous vaginal birth	0.628 (0.161–2.440)	0.501		
Previous cesarean delivery (Y/N)	0.070 (0.08–0.612)	**0.016**	0.118 (0.007–1.891)	0.131
Number of previous cesarean delivery	0.430 (0.141–1.314)	0.139		
Previous abortion/terminations of pregnancy ≥ 2	0.472 (0.121–1.848)	0.281		
Previous myoma enucleation	1.500 (0.312–7.204)	0.613		
No previous cesarean delivery or myoma enucleation	0.114 (0.012–1.069)	0.057		
Multiple pregnancy	0.196 (0.036–1.078)	0.061		
Placenta accreta spectrum	8.800 (0.935–82.804)	0.057		
TOLAC	**6.540 (2.586–16.540)**	**< 0.001**	**7.429 (1.440–38.320)**	**0.017**
Use of oxytocin	2.888 (1.073–7.774)	**0.036**	0.882 (0.179–4.351)	0.878
Use of prostaglandin	1.964 (0.118–32.591)	0.638		

*p*-values < 0.05 were considered significant

After univariate analysis parity > 3, previous vaginal birth, no previous cesarean delivery, TOLAC and use of oxytocin were associated with a more frequently occurrence of CUR in comparison to PUR. After multivariate analysis TOLAC remained the only significant factor

Cases with PUR had with 98.2% (*n* = 55) significantly higher previous cesarean delivery rates compared to 79.3% (*n* = 23) in cases with CUR (*p* = 0.006). In general nearly all cases of PUR occurred in a scarred uterus, whereas CUR was also common in an unscarred uterus (*p* = 0.044, Table 1). In case of PUR, a cesarean delivery was performed in 41 cases (73.2%) without a prior TOLAC and in 15 cases (26.8%) with prior unsuccessful TOLAC. Nearly half of the cases with PUR (*n* = 27, 48.2%) were identified during an elective cesarean. The majority of PUR (*n* = 35, 62.5%) occurred before the onset of uterine contraction.

Obstetric variables and outcomes according to the intended mode of delivery, TOLAC versus ERCD, are shown in Table 5. In the TOLAC group, women with CUR had a significant higher mean number of previous vaginal deliveries (CUR, 1.8 ± 0.2; PUR, 1.0 ± 0; *p* = 0.040).

**Table 5:** Demographic and obstetric factors and outcomes compared between cases with TOLAC and ERCD

	TOLAC (*n* = 30)			ERCD (*n* = 29)			*p* value*
	PUR (*n* = 15)	CUR (*n* = 15)	*p* value	PUR (*n* = 26)	CUR (*n* = 3)	*p* value	
Maternal age ≥ 35 years, *n* (%)	4 (26.7)	5 (33.3)	1.000^2^	9 (34.6)	2 (66.7)	0.539^2^	0.520^1^
BMI > 30 kg/m^2^, *n* (%)	6 (42.9)	6 (46.2)	0.863^1^	5 (19.2)	2 (66.7)	0.136^2^	0.109^1^
Parity ≥ 3, *n* (%)	0 (0)	4 (26.7)	0.100^2^	3 (11.5)	1 (33.3)	0.371^2^	1.000^2^
Previous vaginal birth, *n* (%)	3 (20.0)	5 (33.3)	0.682^2^	3 (11.5)	1 (33.3)	0.371^2^	0.219^1^
Number of previous vaginal births, mean ± SD	1.0 ± 0	1.8 ± 0.2	**0.040**^**4**^	1.0 ± 0	–	–	0.097^4^
Previous cesarean delivery, *n* (%)	15 (100)	15 (100)	^**5**^	26 (100)	3 (100)	^**5**^	–
Number of previous cesarean deliveries. mean ± SD	1.07 ± 0.67	1.0 ± 0	0.317^4^	1.46 ± 0.138	1.33 ± 0.456	0.865^4^	**0.002**^**4**^
Previous VBAC, *n* (%)	1 (6.7)	3 (20.0)	0.598^2^	0 (0)	0 (0)	^**5**^	0.112^2^
Previous myoma enucleation, *n* (%)	0 (0)	0 (0)	^**5**^	3 (11.5)	0 (0)	1.000^2^	0.112^2^
Multifetal pregnancy, *n* (%)	0 (0)	0 (0)	^**5**^	0 (0)	1 (33.3)	0.103^2^	0.492^1^
Gestational diabetes mellitus, *n* (%)	2 (13.3)	1 (9.1)	1.000^2^	5 (20.0)	0 (0)	1.000^2^	0.706^1^
Placenta accreta spectrum, *n* (%)	0 (0)	1 (6.7)	1.000^2^	0 (0)	1 (33.3)	0.103^2^	1.000^1^
Gestational age in weeks, median (range)	41 (35–42)	40 (24–42)	0.237^4^	39 (34–44)	39.5 (38–41)	0.466^4^	** < 0.001**^**4**^
Induction of labor, *n* (%)	2 (13.3)	4 (26.7)	0.651^2^				
With prostaglandins,* n *(%)	*1 (50.0)*	*1 (25.0)*					
with oxytocin,* n *(%)	*1 (50.0)*	*3 (75.0)*		–	–	–	–
Augmentation with oxytocin, *n* (%)	10 (66,7)	8 (53.3)	0.456^1^	–	–	–	–
Regional anesthesia, *n* (%)	14 (93.3)	13 (86.7)	1.000^2^	21 (84.0)	2 (66.7)	0.459^2^	0.464^2^
Successful VBAC, *n* (%)	0 (0)	1 (6.7)	^**5**^	–	–	–	
Pathological CTG, *n* (%)	13 (86.7)	8 (53.3)	0.109^2^	3 (12.0)	2 (66.7)	0.073^2^	** < 0.001**^**1**^
Uterine atony, *n* (%)	0 (0)	4 (26.7)	0.102^2^	1 (4.8)	1 (33.3)	0.239^2^	0.674^2^
Symptomatic uterine rupture, *n* (%)	14 (93.3)	14 (93.3)	1.000^2^	7 (28.0)	2 (66.7)	0.234^2^	** < 0.001**^**1**^
Maternal outcome							
Postpartum hysterectomy, *n* (%)	0 (0)	0 (0)	^**5**^	0 (0)	1 (33.3)	0.103^2^	0.492^2^
Puerperal complications,* n* (%)	5 (33.3)	10 (66.7)	0.068^1^	12 (46.2)	1 (33.3)	1.000^2^	0.691^1^
*Fever* > *2d* > *38 °C postpartum, n (%)*	*0 (0)*	*2 (20.0)*		*2 (16.7)*	*0 (0)*		
*Impaired wound healing, n (%)*	*0 (0)*	*1 (10.0)*		*2 (16.7)*	*0 (0)*		
*Hypertension, n (%)*	*0 (0)*	*1 (6.7)*		*0 (0)*	*1 (100)*		
*Anemia, n (%)*	*5 (100)*	*9 (60.0)*		*8 (66.7)*	*1 (100)*		
Maternal blood transfusion, *n* (%)	0 (0)	3 (20.0)	0.224^2^	1 (3.8)	1 (33.3)	0.200^2^	1.000^2^
Maternal death, *n* (%)	0 (0)	0 (0)	^**5**^	0 (0)	0 (0)	^5^	^5^
Neonatal outcome^a^							
Male infant, *n* (%)	8 (53.3)	9 (60.0)	0.713^1^	17 (65.4)	2 (100)	1.000^2^	0.380^1^
Birth weight ≥ 3500 g, *n* (%)	9 (60.0)	8 (53.3)	0.713^1^	7 (26.9)	1 (50.0)	0.497^2^	**0.031**^**1**^
Perinatal death, *n* (%)	0 (0)	2 (14.3)	0.224^2^				
*Mortality before hospital admission, n (%)*		*1 (50.0)*		*0 (0)*	*0 (0)*	^5^	0.491^2^
cord blood pH < 7.2, *n* (%)	2 (13.3)	8 (57.1)	**0.021**^**2**^	1 (3.8)	0 (0)	1.000^2^	**0.003**^**1**^
cord blood pH < 7.0, *n* (%)	0 (0)	4 (28.6)	**0.042**^**2**^	0 (0)	0 (0)	^5^	0.112^2^
5-min Apgar score < 7, *n* (%)	0 (0)	2 (14.3)	0.224^2^	1 (3.8)	1 (50.0)	0.140^2^	1.000^2^
Need of intensive care,* n* (%)	1 (6.7)	5 (35.7)	0.080^2^	3 (11.5)	1 (50.0)	0.270^2^	0.525^1^
Hypoxic–ischemic encephalopathy *n* (%)	0 (0)	0 (0)	^5^	0 (0)	0 (0)	^5^	^5^

*p*-values < 0.05 were considered significant

^1^Chi square test

^2^Fisher’s exact test

^3^Student *t* test

^4^Mann–Whitney *U* test

^5^Calculation not possible

^*^p value comparing TOLAC and ERCD regardless of type of uterine rupture

^a^After ERCD: *n* = 28 (one case of triplets excluded)

Due to restrictive use of prostaglandins and oxytocin for labor induction in our clinic only 20% (n = 6) of the women undergoing TOLAC had induction of labor, 2 patients (6.6%) received prostaglandins and 4 patients (13.3%) oxytocin and further 18 patients (60%) had oxytocin support during labor. There were no significant differences between CUR and PUR regarding labor induction and augmentation.

In women undergoing TOLAC, the CUR rate was with 50% (*n* = 15) significantly higher compared to ERCD with 10.3% (*n* = 3; *p* = 0.001). Nevertheless, no differences regarding maternal outcomes were observed.

As expected, neonatal acidosis was seen more often in 34.5% (*n* = 10) after TOLAC compared to 3.6% (*n* = 1) after ERCD (*p* = 0.003). In addition, when comparing between PUR und CUR in women with TOLAC, neonatal acidosis was significant more frequent in women with CUR (CUR, *n* = 8, 57.1%; PUR, *n* = 2, 13.3%; *p* = 0.021). There were no significant differences regarding the 5-min APGAR, the need of intensive care or the occurrence of hypoxic–ischemic encephalopathy and perinatal death.

## Discussion

To date, identifying patients at risk of uterine rupture remains challenging. Since CURs are associated with poorer maternal and fetal outcomes, several studies have investigated risk factors for uterine rupture but with no distinction between the types or only under the consideration of complete or symptomatic uterine ruptures. Only few distinguished between complete and partial uterine rupture, which will lead to an artificial selection of patients and bias in the study results [2, 5, 19–22]. As a result, associated factors with the occurrence of PUR have rarely been studied so far. Moreover, there could be a substantial loss of information, especially since in our study, collective 66% of all cases with uterine ruptures were partial (PUR). Our study confirmed that the outcome between CUR and PUR is different, and therefore, it is important to distinguish between them.

In line with others and as expected, we found that CURs were significantly more frequent after TOLAC compared to ERCD [6]. Furthermore, our study confirmed that cases with CUR were associated with worsened maternal and fetal outcomes. After multivariate regression analysis, TOLAC was the only independent risk factor for CUR in our study. As a result, early and standardized evaluation for PUR in women with previous cesarean section prior to a planned TOLAC is essential. Currently the role of sonographic evaluation of LUS after cesarean delivery and its clinical benefit in assessing the risk of scar dehiscence are still controversial [23]. Nevertheless, some studies provide evidence that uterine scar assessment may be a useful tool for early identification of patients at risk [24, 25].

In general, the route of delivery after cesarean section is widely discussed. When offering women ERCD several risks have to be taken into account: high risks of short-term complications such as hemorrhage, hysterectomy, thromboembolism, and neonatal complications that include respiratory distress syndrome and long-term complications such as placenta previa and accreta in future pregnancies [7–10, 26, 27]. Nevertheless, contraindications for TOLAC on the other hand such as previous uterine rupture, previous fundal incision or a present abnormally invasive placenta must be considered [28]. When analysing the intended route of delivery in our study, as expected we found that CURs were significantly more prevalent in cases with TOLAC compared to ERCD.

In patients counselled for TOLAC versus ERCD, interestingly neonatal outcomes, such as Apgar scores, admission to the neonatal intensive care unit and perinatal mortality, were in our study in general comparable between TOLAC and ERCD. Only neonatal acidosis rates were significantly higher after TOLAC, especially in case of CUR, compared to ERCD.

It is well known that compared with ERCD, TOLAC per se increases the risk of uterine rupture to 2.7 per 1000 cases, on the other hand 370 elective cesarean deliveries would be needed to prevent one symptomatic uterine rupture [29, 30]. The maternal outcome, including low hysterectomy rates, and fetal outcome, except for neonatal acidosis, were comparable between ERCD and TOLAC at our university hospital despite occurrence of uterine rupture. Thus, the data suggest that TOLAC can be performed relatively safely in a high resource setting after the mother got advised about the advantages and disadvantages of the two possible birth modes, provided there are no contraindications.

Previous vaginal births are often cited as a marker for a successful TOLAC [14, 31, 32]. Interestingly, in our study cohort in the TOLAC-group women with a higher number of previous vaginal births had a higher risk for CUR compared to PUR. Therefore, it should be considered that even a high number of previous vaginal births does not eliminate the risk of uterine rupture and, in case of uterine rupture, leads primarily to CURs.

Almost all uterine ruptures during TOLAC were symptomatic leading to faster diagnosis and therapy, whereas with ERCD only one third of the cases were symptomatic. Therefore, in patients undergoing TOLAC the delivery mode must be reconsidered as soon as clinical signs such as pain in LUS, maternal hypotension or a pathologic CTG occur. However, it is important to notice that uterine rupture can be preceded or accompanied by several types of changes in uterine contractility including hyperstimulation, reduced number of contractions and increased or reduced baseline of the uterine tonus, while no typical pattern has been repeatedly reported and, therefore, remains unspecific [33]. Furthermore, a pathological CTG is common in women with TOLAC and not a strong predictor for threatening uterine rupture [34].

In line with Guiliano et al. and others we observed ruptures of the unscarred uteri were more frequently CURs with worsened maternal and neonatal outcomes [17, 35, 36]. Markou et al. found that ruptures of the unscarred uterus are associated with significantly more maternal and fetal complications [19]. Risk factors for spontaneous CUR have not been clearly identified yet. One study with 20 cases of CUR of the unscarred uteri showed an association with multiparity, epidural analgesia and augmentation by oxytocin [37]. In our study cohort four out of five ruptures of the unscarred uteri were CURs. A possible explanation for this could be that a rupture that occurs on an unscarred uterus must be triggered by a very strong force, which then directly promotes a CUR. In our collective multiparity ≥ 3 was present in most cases with a spontaneous rupture.

Induction of labor with prostaglandins in women with TOLAC increases the risk for uterine rupture further [38]. A possible explanation for the higher risk of uterine rupture associated with prostaglandin usage is that those might induce ultrastructural changes that weakens the scar [39]. In our study the number of patients who received induction of labor either with prostaglandins or oxytocin was too limited to draw a conclusion. A meta-analysis of the use of oxytocin in the induction of labor or augmentation demonstrated that the pooled rate of uterine rupture of women using oxytocin was 1.4% in comparison to 0.5% in women not using oxytocin (*p* = 0.0002) [40]. The use of any oxytocin in our study for induction or augmentation was associated with increased risk for CUR, but in the subgroup analyses when we examined the use of oxytocin separately either only for induction or for augmentation during labor, there were no significant differences. Our results allow the hypothesis that when oxytocin is used in a low dose with a maximum of 0.48 I.E./h as in our clinic protocol for labor augmentation, it does not increase significantly the risk for CUR.

Several studies have identified risk factors, but most study designs are inhomogeneous, since no general definition of uterine ruptures was made [5, 20, 41, 42]. To notice, in our study no differences in regard to demographic risk factors such as maternal age ≥ 35 years, height ≤ 160 cm and BMI > 30 kg/m^2^ between cases with CUR and PUR were found, indicating that those generally contribute to the occurrence of uterine rupture without having an influence on the type of rupture. Recently, Antila-Langsjö et al. revealed that maternal BMI, gestational diabetes, and previous cesarean deliveries are associated with an increased risk for incomplete healing of the uterine incision [43]. Our study confirmed these findings showing overall obesity rates of 31.7% and high previous cesarean delivery rates (91.7%). The gestational diabetes rate was comparatively low with 11.8%.

Limitations are the retrospective nature of this study, the small patient cohort when analysing subgroups and the long study period of 13 years at a single centre. Nevertheless, although incidence rates are increasing uterine ruptures remain a rare peripartum complication and so prospective observational studies are challenging. Furthermore, a total of 7 cases with uterine rupture were excluded from this analysis, because, due to the retrospective data, no distinction between CUR and PUR could be made. Another potential selection bias is that cases with asymptomatic uterine rupture and vaginal delivery may have been missed. Furthermore, the study was a monocentric study by a high-quality emergency obstetrical care facility in a high-resource setting, so that results might be only transferable to centres with comparable medical resources.

In conclusion, this study assessed risk factors and outcomes associated with the type of uterine rupture and the intended route of delivery retrospectively. We found that TOLAC is the only independent risk factor for CURs, which is associated with significantly worsened maternal and fetal outcomes. After optimized antenatal counselling of patients for the intended route of delivery, independent of the type of rupture, TOLAC and ERCD showed comparable short-term maternal and fetal outcomes in a high resource setting. In women undergoing TOLAC a high number of previous vaginal births does not eliminate the risk of uterine rupture and, in case of uterine rupture, leads primarily to CUR. Due to different influencing factors and outcomes, we are in need of a standardized definition of both PUR and CUR to ensure comparability between studies.

## Supplementary Information

Below is the link to the electronic supplementary material.

Supplementary file1 (DOCX 17 KB)

## Abbreviations

**ACOG**: American College of obstetricians and gynecologists
**CTG**: Cardiotocography
**CUR**: Complete uterine rupture
**ERCD**: Elective repeat cesarean delivery
**GerOSS**: German obstetric surveillance system
**LUS**: Lower uterine segment
**PUR**: Partial uterine rupture
**TOLAC**: Trial of labor after cesarean delivery
**VBAC**: Vaginal birth after cesarean delivery
