# Supplementary material for: Risk factors and outcomes associated with type of uterine rupture
Source: Arch Gynecol Obstet. 2022 Mar 14;306(6):1967–77. doi: 10.1007/s00404-022-06452-0 (PMC9633527; doi:10.1007/s00404-022-06452-0)
Supplement: Supplementary file 1 — Supplementary file1 (DOCX 17 KB) [file 404_2022_6452_MOESM1_ESM.docx]

**Supplemental table**

**ST 1 – Cases with spontaneous uterine rupture**

| **Number**  **Type of uterine rupture** | **1**  **CUR** | **2**  **CUR** | **3**  **CUR** | **4**  **CUR** | **5**  **PUR** |
| --- | --- | --- | --- | --- | --- |
| Age | 39 | 43 | 31 | 34 | 39 |
| BMI mother (kg/m^2^) | 31 | 23 | NK | 24 | 28 |
| Multifetal pregnancy | No | No | No | No | Yes |
| Gravidity/Parity | 4/4 | 7/4 | 5/4 | 5/3 | 1/1 |
| Previous miscarriage (Number) | No | Yes (2) | Yes (1) | Yes (2) | No |
| Previous termination of pregnancy (Number) | No | Yes (1) | No | No | No |
| Previous curettages (number) | No | Yes (3) | NK | Yes (1) | No |
| Gestational diabetes mellitus | No | No | No | No | Yes |
| Gestational age in weeks | 38+6 | 40+4 | 40+0 | 38+4 | 36+1 |
| Induction of labor | No | No | No | No | No |
| Prostaglandin use | No | No | No | No | No |
| Oxytocin use (dose in I.E.) | Yes (0,35) | No | No | No | No |
| Peridural anesthesia | Yes | No | No | Yes | Yes |
| Cervical opening (cm) | 9 | 10 | 2 | 5 | 0 |
| Pathological CTG | Yes | No | Yes | Yes | No |
| Placenta accreta spectrum | No | Yes (accreta) | No | No | No |
| Hysterectomy | Yes | No | Yes | No | No |
| Blood loss (ml) | 4000 | 1500 | 2000 | 1000 | 1000 |
| Maternal blood transfusion | Yes | Yes | Yes | No | No |
| Birth weight in g | 4140 | 3730 | 2690 | 3020 | 2955  2535 |
| APGAR 1/5/10min | 2/6/7 | 8/9/10 | 8/9/9 | 9/9/10 | 9/10/10  9/10/10 |
| Cord blood pH | 6,79 | 7,29 | 7,26 | 7,18 | 7,28  7,25 |
| NICU | Yes | No | No | No | No |
| Peripartum death | No | No | No | No | No |

CUR= complete uterine rupture, NICU= neonatal intensive care unit

NK= not known, PUR= partial uterine rupture
